# ASO Author Reflections: Practice, Preference, and Price: Reflections on Alpha-Blockade Choices

**DOI:** 10.1245/s10434-026-19477-5

**Published:** 2026-03-25

**Authors:** Lauren R. Kelz, Heather Wachtel

**Affiliations:** Department of Surgery, University of Pennsylvania, Philadelphia, USA

## Past

Alpha-blockade is the standard of care in the preoperative management of pheochromocytoma.^1,2^ Nonselective alpha-blockade was historically predominant. However, in recent years, the greater accessibility and lower cost of selective alpha-blockade have led to a shift in practice, particularly as medication costs often represent out-of-pocket expenses for patients.^3^ Although prior studies suggest comparable clinical outcomes between selective and nonselective blockade, little is known about whether these approaches differ in their impact on perioperative resource utilization and overall costs across the episode of care.^4^ We therefore sought to evaluate the comparative costs of selective and nonselective alpha-blockade across the continuum of perioperative care.

## Present

We evaluated a national cohort of patients who underwent adrenalectomy for pheochromocytoma from Optum’s de-identified Clinformatics^®^ Data Mart Database.^5^ The primary exposure was alpha-blockade type, stratified by selective (prazosin, doxazosin, terazosin) and nonselective (phenoxybenzamine). As expected, selective blockade was associated with lower medication costs in the 30 days preceding surgery. Importantly, however, there were no significant differences in downstream expenditures, including costs from admission to discharge, costs in the 30 days following discharge, cumulative perioperative costs, or length of stay. In short, overall costs and short-term outcomes were comparable between blockade strategies.

## Future

Our findings suggest that selective alpha-blockade does not lead to increased downstream costs—put simply, “cheaper isn’t worse.” These results support the use of selective alpha-blockade, particularly when medication costs may pose a financial burden to patients. Future work should quantify patient-level out-of-pocket expenses to better understand the financial implications of preoperative management decisions. Additionally, correlating outcomes with biochemical tumor burden may help clarify whether catecholamine secretion levels should inform blockade selection. Prospective studies will be critical to further refine cost-conscious, patient-centered perioperative care for pheochromocytoma.
